# The gut bacterial community affects immunity but not metabolism in a specialist herbivorous butterfly

**DOI:** 10.1002/ece3.6573

**Published:** 2020-07-16

**Authors:** Anne Duplouy, Guillaume Minard, Marjo Saastamoinen

**Affiliations:** ^1^ Department of Biology, Biodiversity Unit Lund University Lund Sweden; ^2^ Research Centre for Ecological changes, Organismal and Evolutionary Biology Research Program Faculty of Environmental and Biological Sciences University of Helsinki Helsinki Finland; ^3^ Laboratory of Microbial Ecology UMR CNRS 5557 UMR INRA 1418 University Claude Bernard Lyon 1 Villeurbanne France; ^4^ Helsinki Institute of Life Science University of Helsinki Helsinki Finland

**Keywords:** antibiotic treatment, gut microbial community, immunity, larval development, larval survival, metabolites

## Abstract

1. Plant tissues often lack essential nutritive elements and may contain a range of secondary toxic compounds. As nutritional imbalance in food intake may affect the performances of herbivores, the latter have evolved a variety of physiological mechanisms to cope with the challenges of digesting their plant‐based diet. Some of these strategies involve living in association with symbiotic microbes that promote the digestion and detoxification of plant compounds or supply their host with essential nutrients missing from the plant diet. In Lepidoptera, a growing body of evidence has, however, recently challenged the idea that herbivores are nutritionally dependent on their gut microbial community. It is suggested that many of the herbivorous Lepidopteran species may not host a resident microbial community, but rather a transient one, acquired from their environment and diet. Studies directly testing these hypotheses are however scarce and come from an even more limited number of species.
2. By coupling comparative metabarcoding, immune gene expression, and metabolomics analyses with experimental manipulation of the gut microbial community of prediapause larvae of the Glanville fritillary butterfly (*Melitaea cinxia*, L.), we tested whether the gut microbial community supports early larval growth and survival, or modulates metabolism or immunity during early stages of development.
3. We successfully altered this microbiota through antibiotic treatments and consecutively restored it through fecal transplants from conspecifics. Our study suggests that although the microbiota is involved in the up‐regulation of an antimicrobial peptide, it did not affect the life history traits or the metabolism of early instars larvae.
4. This study confirms the poor impact of the microbiota on diverse life history traits of yet another Lepidoptera species. However, it also suggests that potential eco‐evolutionary host‐symbiont strategies that take place in the gut of herbivorous butterfly hosts might have been disregarded, particularly how the microbiota may affect the host immune system homeostasis.

## Graphical abstract

Our study showed that the microbiota within the gut of Lepidoptera could efficiently be altered by antibiotic treatment and restored either by fecal transplants or self‐resilience after antibiotic relaxation. The dominant species of the gut bacterial community of the larvae did not impact larval performances and metabolism, but rather significantly impacted the host immunity by up‐regulating the expression of a gene involved in the response against pathogens.

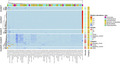

## 1 INTRODUCTION

Herbivory results in the extraction and assimilation of nutrients and energy from a plant diet. This adaptation supports the development and survival of many vertebrates and invertebrates on Earth. Plant tissues might, however, be of low nutritious value, and many are rich in toxic defensive compounds. In insects, many herbivores are generalists and feed on a wide range of plants that provide a large diversity of nutrients (Hagele & Rowell‐Rahier, 1999). Specialist herbivores, on the other hand, have evolved a range of adaptive behavioral, physiological, and anatomical strategies to optimize their nutrient intakes, and consequently their fitness, from a small range of host plant species (Lampert, 2012; Lampert & Bowers, 2010). Some of these strategies call for dependence upon symbiotic associations with microorganisms colonizing the guts or other specialized organs of the hosts (Brune, 2014; Douglas, 1998; Hosokawa, Koga, Kikuchi, Meng, & Fukatsu, 2010). These microorganisms, often but not only, bacteria and fungi, can directly provision their host with nutrients lacking from their restricted plant‐based diet or facilitate the digestion of various plant compounds. For example, in aphids, the endosymbiotic bacteria *Buchnera aphidicola* provide essential amino acids that are generally absent from the phloem sap diet of their host plant (Hansen & Moran, 2011; McCutcheon & Moran, 2012; Poliakov et al., 2011). Similarly, lower termites rely upon protozoans and bacteria colonizing their guts to digest the lignocellulose of wood into nutritious fatty acids (Bandi & Sacchi, 2000; Nazarczuk, Obrien, & Slaytor, 1981; Slaytor & Chappell, 1994). This direct nutritional impact on individuals fitness is further translated in the long‐term impact on the fitness of the entire colony, including colony longevity, colony growth rate, and queen fertility (Rosengaus, Zecher, Schultheis, Brucker, & Bordenstein, 2011).

A growing body of evidence is supporting the idea that many Lepidoptera species rarely carry beneficial symbionts within their microbiota (Hammer, Janzen, Hallwachs, Jaffe, & Fierer, 2017). Furthermore, although the diversity and absolute number of microorganisms evolving in the soil environment and on host plant surfaces have been shown to be generally high (Whitaker, Salzman, Sanders, Kaltenpothz, & Pierce, 2016), the diversity and absolute number of microorganisms colonizing the gut of Lepidoptera species evolving in these environments are much lower (Hammer et al., 2017; Vilanova, Baixeras, Latorre, & Porcar, 2016). More than 60% of the gut microbiota of Lepidoptera species is represented by only ten phyla, often dominated by bacterial species from the *Pseudomonas*, *Bacillus*, *Enterococcus*, and *Staphylococcus* genera (Hammer et al., 2017; Vilanova et al., 2016; Voirol, Frago, Kaltenpoth, Hilker, & Fatouros, 2018). Despite studies suggesting that most part of those microbial communities are potentially acquired from the host environment, and diet, we still lack experimental studies directly testing these hypotheses. Additionally, evidence of the poor contribution of the gut microbiota to the insect nutritional intake yet only come from a limited number of species (e.g., in *Lycaenid* butterflies (Chaturvedi, Rego, Lucas, & Gompert, 2017), and others (Hammer et al., 2017)), and consequently many potential eco‐evolutionary host‐symbiont strategies that take place in the gut of herbivorous Lepidopteran hosts have yet to be described.

To thoroughly address whether the gut microbiota matters to the development, survival, and metabolism of larvae of specialist herbivore butterflies, we empirically set up an experiment to disrupt and restore the microbiota of early instar larvae through antibiotic treatments followed by fecal transplants from conspecifics. We used the Glanville fritillary butterfly (*Melitaea cinxia*, Linnaeus 1758) as previous laboratory experiments showed that the performance of the larvae from local populations of this species varies between the host plants, and between plants of different quality (Laine, 2004; Rosa, Woestmann, Biere, & Saastamoinen, 2018; Salgado & Saastamoinen, 2019; Van Nouhuys, Singer, & Nieminen, 2003). Two recent studies also suggest a correlation between the composition of the larval microbiota of the Glanville fritillary butterfly and different aspects of the species fitness, including larval growth and performances (Rosa, Minard, Lindholm, & Saastamoinen, 2019; Ruokolainen, Ikonen, Makkonen, & Hanski, 2016). However, and as it is often the case for this kind of study, these former two studies have failed to provide some critical controls to the microbiota experiments through not testing for the effect of experimental manipulation of the gut microbial community composition (Rosa et al., 2019; Ruokolainen et al., 2016). As the microbiota may also be environmentally acquired, we thus also evaluated the self‐resilience of the microbiota by adding one treatment group during which the antibiotic treatment was maintained during fecal transplant. The efficiency of this protocol was assessed by metabarcoding of the bacterial communities associated to the gut of the larvae. The community resilience of re‐infected individuals was estimated based on the community structure of antibiotic‐treated and nontreated individuals. Finally, we were particularly interested in testing whether the manipulation of the gut microbiota affected larval performances, by analyzing variation in life history traits (i.e., larval development and survival), immunity, and metabolism among the treatment groups.

## 2 MATERIALS AND METHODS

### 2.1 Larvae and plant material

The Glanville fritillary butterfly, *Melitaea cinxia*, L. 1758, is a widely distributed species across Eurasia and North Africa. Over the last three decades, many aspects of the ecology, life history, demography, and eco‐evolutionary dynamics of the Finnish population inhabiting the Åland islands, in the Baltic Sea, have been intensively studied (Duplouy, Ikonen, & Hanski, 2013; Hanski, 2011; Nieminen, Siljander, & Hanski, 2004). Across the entire European range of this butterfly, the larvae feed on plant species from only two genera, *Plantago* and *Veronica* (Kuussaari & Singer, 2017). Our laboratory stock population was originally assembled by collecting three individual larvae from 38 (F0) larval families across the Åland metapopulation in September 2015, as previously described (Rosa et al., 2018). Although larvae were not genotyped, our sampling strategy insured that we worked on representative individuals from the genetic diversity of the metapopulation (Fountain et al., 2016; Nair, Fountain, Ikonen, Ojanen, & van Nouhuys, 2016). Larvae were reared in larval family groups under optimum conditions (during periods of growth: Day:Night (D:N), 27°C:10°C, 12 hr:12 hr; during diapause: D:N, 5°C:5°C, 12 hr:12 hr) over two generations at the Lammi Biological station, University of Helsinki, Finland. Butterflies were mated with nonsibling partners (from a different larval family) in the laboratory, and mated females were individually isolated in small cages to lay eggs on *Plantago lanceolata* host plants. The host plants were checked daily for egg clutches, which were carefully transferred into individually labeled petri dishes.

On the day of emergence from the eggs, L1 (F2) larvae from each of 27 selected larval families (*N*_Total_ = 2,160) were divided between four treatments in groups of 20 individuals each in a full factorial design (Family × Treatment, to allow for testing the effect on each response variable of family, treatment, and their interaction independently) and reared in the laboratory until 3rd instar (L3), as described below. In parallel, we also reared postdiapause (F1) larvae from five larval families under optimum laboratory conditions (above) and collected fresh frass every day once the larvae reached 7th instar (L7). Sample sizes for each treatment and experiment are described in Table 1.

**TABLE 1:** Sample size for each treatment and each experimental assay

Sample type and treatment		Development and Survival	Microbiota	Gene expression	Metabolomics
C	Control larvae (No treatment)	20 larvae × 27 families	3 guts × 13 families	3 larvae × 10 families + (2 larvae × 1 family) + (1 larva × 1 family)	3 carcasses pooled × 13 families
A	Antibiotics from emergence	20 × 27	3 × 13	3 × 10 + (2 × 1) + (1 × 1)	3 pooled × 13
AR	Antibiotics from emergence + L7 frass on day4	20 × 27	3 × 13	3 × 12	3 pooled × 13
R	Antibiotics until day3 + L7 frass on day4	20 × 27	3 × 13	3 × 9 + (2 × 1) + (1 × 2)	3 pooled × 13
Frass (10 mg)		–	5	–	2
Plant leave (10 mg)		–	5	–	1
Control (sterile water)		–	5	3	–
Total (*N*=)		2,160	171	136	55

Note

All assays were run individually for each sample but the metabolomics for which the carcasses of three larvae of the same larval family were pooled. L3 and L7: third and seventh instar larvae, respectively. Treatments are colored following the color code used in the study figures.

*Plantago lanceolata* (*N* = 120) was used as the larval food throughout the experiment (see below). The plants were collected as seeds across the Åland islands in 2015 and grown in optimum laboratory greenhouse conditions at the Lammi Biological Research station (D:N, 27°C:10°C, 12 hr:12 hr). Plants were watered every 3rd day. Plant leaves were only harvested for the experiment, thus preserving all natural defensive metabolites and original microbial load of the plants for the experiment. We also harvested and froze in liquid nitrogen some extra leaves to provide controls to the experiments described below.

### 2.2 Treatments

**FIGURE 1:**
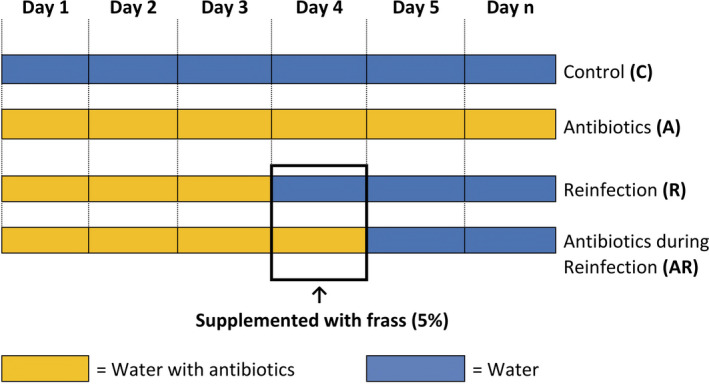
Daily description of the four larval treatment groups used in the study. (C) A daily amount of 200 µl of sterile water was left to dry 2 hr on the leaves before being provided to the larvae, from day1 (L1) until the larvae molt into L3. (A) 200 µl of the antibiotic solution was left to dry 2 hr on the leaves before being provided to the larvae, from day1 (L1) the larvae molt into L3. (R) 200 µl of antibiotic solution was left to dry 2 hr on the leaves before being provided to the larvae, from day1 to day3 (L1). On day 4, 200 µl of the antibiotic solution was supplemented with 5% of L7's frass and left to dry 2 hr on the leaves before being provided to the larvae. From day5 and until the larvae molted into L3, 200 µl of sterile water was left to dry 2 hr on the leaves before being provided to the larvae. (AR) 200 µl of the antibiotic solution was left to dry 2 hr on the leaves before being provided to the larvae, from day1 to 3 (L1). On day4, 200 µl of the antibiotic solution was supplemented with 5% of L7's frass and left to dry 2 hr on the leaves before being provided to the larvae. From day5, 200 µl of sterile water was left to dry 2 hr on the leaves before being provided to the larvae until the larvae molt into L3

The 2,160 larvae from 27 larval families were equally divided between four treatments. Each group of 20 larvae was given daily a freshly harvested 1.7 cm^2^ piece of randomly collected *P. lanceolata* leaf (Minard, Tikhonov, Ovaskainen, & Saastamoinen, 2019), which was supplemented differentially according to treatment (Figure 1):

- (**C**ontrol): 200 µl of sterile water was left to dry on the leaves before being provided to the larvae, from day1 (L1) until the larvae molt into L3.
- (**A**ntibiotic): 200 µl of the antibiotic solution was left to dry on the leaves before being provided to the larvae, from day 1 (L1) the larvae molt into L3.
- (**R**e‐infection): 200 µl of antibiotic solution was left to dry on the leaves before being provided to the larvae, from day1 to day3 (L1). On day4, 200 µl of sterile water supplemented with 5% of L7's frass was left to dry on the leaves before being provided to the larvae. From day5 and until the larvae molted into L3, 200 µl of sterile water was left to dry on the leaves before being provided to the larvae.
- (**A**ntibiotic during **R**e‐infection): 200 µl of the antibiotic solution was left to dry on the leaves before being provided to the larvae, from day1 to 3 (L1). On day4, 200 µl of the antibiotic solution was supplemented with 5% of L7's frass and left to dry on the leaves before being provided to the larvae. From day5, 200 µl of sterile water was left to dry on the leaves before being provided to the larvae until the larvae molt into L3.

The antibiotic solution was prepared by mixing three antibacterial agents (2 × 10^−4^ g/ml of neomycin sulfate, with 1 × 10^−3^ g/ml of aureomycin, 6 × 10^−5^ g/ml of streptomycin) and two antifungal agents (8 × 10^−4^ g/ml of methyl paraben, and 6 × 10^−4^ g/ml of sorbic acid) as described by Chung, Rosa, Hoover, Luthe, and Felton (2013).

### 2.3 Larval performance: Development and survival

For each larval group, transition to the 2nd larval instar was checked every day over a 13 day long period, while survival until 3rd larval instar within each group was estimated every third day in that same 13^‐^day long period. On the day the surviving larvae reached the 3rd instar, they were frozen in liquid nitrogen and stored at −80°C until further manipulated. As the larvae were not starved before being killed, the gut content of most larvae may still include material from the diet. Due to the large number of larval families and treatment groups, not all larval groups could be reared during the exact same days; instead, the emergence dates of the larvae from the eggs spread over eight successive days. The larvae from the same larval family all emerged on the same day. Larvae were reared at 23°C with lights on between 8:00–10:00 a.m., and between 3:00 p.m. and 5:00 p.m., at 28°C with lights on between 10:00 a.m. and 3:00 p.m., and at 18°C in the dark between 5:00 p.m. and 8:00 a.m.

### 2.4 Metabarcoding of the gut microbiota

We surface sterilized three L3 larvae from each of the four treatments for 13 larval families before individually dissecting their gut out under a microscope in a sterile laminar flow hood. All larval carcasses were preserved to perform the metabolomics analyses described below (see *Metabolomics* section). We individually extracted the DNA from the gut of the 156 larvae under sterile conditions. The DNA was extracted using a Qiagen DNeasy Blood and Tissue kit (Qiagen, Germany) following an optimized protocol as described by Minard et al. (2015). Three additional extractions were carried out on sterile water to control for environmental contamination during the procedure. We amplified the hypervariable V5‐V6 bacterial region of the *rrs* gene using the primers 784F (5′‐AGGATTAGATACCCTGGTA) and 1061R (5′‐CRRCACGAGCTGACGAC) (Andersson et al., 2008). Amplification of the LSU region of the *ITS* gene of Ascomycota fungi using the primers LSU200A‐*F* (5′‐AACKGCGAGTGAAGCRG) and LSU476A‐R (5′‐CSATCACTSTACTTGTKC) (Asemaninejad, Weerasuriya, Gloor, Lindo, & Thorn, 2016) did not successfully amplify enough fungal sequences for a comprehensive community analysis, presumably due to the limited fungal community associated with the larvae of the Glanville fritillary butterfly. Each sample was amplified in duplicate and using 3 µl of the DNA extract for each PCR reaction (Minard et al., 2015), and the duplicates for each sample were pooled in sterile condition after amplification. Sequencing was performed by the Institute for Molecular Medicine Finland (FIMM, Finland) on a Miseq v.3. Sequencing platform (Illumina, USA) using both reverse and forward primers.

We analyzed the libraries using *Mothur* v.1.37.6 (http://www.mothur.org/wiki/MiSeq_SOP). (Schloss et al., 2009). We selected all 250‐350bp‐long sequences, with less than eight homopolymers, no ambiguous position, and which aligned to the *rrs* Silva v.123 database. Chimeric sequences were removed using UCHIME implemented in *Mothur* (Edgar, Haas, Clemente, Quince, & Knight, 2011). Sequences were clustered within operational taxonomic units (OTUs) according to average neighbor method with 3% distance maximum within each OTU. All OTUs showing at least a 10× higher proportion in any given sample than in the negative controls were considered as contaminant and removed from our dataset using an in‐house R script (Minard et al., 2019).

### 2.5 Metabolomic analysis of the larval carcasses

We used the carcasses of the larvae used in the microbiota assays described above for the metabolomics analyses. Using only the carcasses without the gut allowed us to provide information of the metabolites from the larvae without contamination from the plant diet. After dissection, the three larval carcasses from each larval family (*N*_total_ = 13 larval families) were pooled and crushed in liquid nitrogen using a sterile pestle. Similarly, we also crushed two samples of 30 mg of *P. lanceolata* leaves each and two samples of 30 mg of larval frass in liquid nitrogen using sterile pestle, to use as controls for diet metabolite that might still contaminate our larval samples. All 55 samples (52 pooled larval carcasses, one host plant and two frass controls) were then freeze‐dried for 48 hr in a freeze dryer (MechaTech Systems Ltd). Dry samples were weighted on an analytical balance (*d* = 0.1 mg, Fisher Scientific, UK). Metabolites were then extracted using the protocol described by Kim et al. (Kim, Choi, & Verpoorte, 2010). In brief, for each sample, we placed 10 mg of dry material in 350 µl of CD_3_OD (VWR Chemicals, Belgium) and 350 µl of KH_2_PO_4_ (Sigma‐Aldrich, Germany) buffer mixed in D_2_O (pH6) containing 0.05% (wt/wt) of TSP (sodium trimethylsilylpropionic acid) (Sigma‐Aldrich, USA). Samples were then sonicated for 20 min and centrifuged 10 min at 17,000 *g*. We transferred 600 µl of the clear supernatants into individual 5 mm diameter NMR tube (Wilmad, USA), and the metabolite content of each sample was analyzed using a Bruker 850 MHz Advance III HD NMR spectrometer equipped with a TCI Cryoprobe (Bruker, USA) at the Finnish Biological NMR Center, the University of Helsinki, Finland.

Proton Nuclear Magnetic Resonance (^1^H‐NMR) spectra were acquired at 298 K and recorded using 1D presaturation pulse sequence (zgpr). For each ^1^H spectrum, 256 transients were collected into 32 K time domain points using a 60° flip angle, spectral width of 10.2 kHz, relaxation delay of 5.0 s, an acquisition time of 1.6 s, and a mixing time of 5 ms. Fourier transformation of the free‐induction decay was applied with zero filling to give 65 K frequency domain data points. The preliminary treatments of the ^1^H‐NMR spectra were performed using the software MNOVA v.10.0.2 (Mestrelab research S.L., Spain). Standard solutions containing 1 mg of one of the five antibiotics used for the treatments were measured individually in order to enable their identification, quantification, and trimming from the antibiotic‐treated larval samples.

### 2.6 Immune gene expression

We individually sampled up to 12 larvae from each of the 12 larval families and flash‐froze them in liquid nitrogen once they had reached the third larval instar. For five of the 12 larval families, we included three L3 larvae for each treatment, while for the remaining seven larval families, we only had one or two larvae for some of the treatments due to mortality during development (*N*_total_ = 133).

The RNA from each larva was individually extracted following a protocol described by Woestmann, Kvist, and Saastamoinen (2017) using TRIzol reagent (Life Technologies Corporation), acid‐phenol:chloroform:isoamyl alcohol (25:24:1, pH = 5), and chloroform. The RNA was then precipitated using isopropanol, washed in 75% ethanol, air‐dried in a flow hood, and re‐suspended in 50 µl MQ water. Potential genomic DNA contaminants were removed using DNase I (Thermo Fisher Scientific Inc.). The RNA was reverse‐transcribed using an iScript™ cDNA Synthesis Kit (Bio‐Rad Laboratories) following the manufacturer's protocol.

The qPCR assays were performed with three technical replicates for each sample, and one negative control and plate control (same sample across all plates) for each 384‐well plate used, in a 10 µl volume, on a C1000™ Thermal Cycler (Bio‐Rad Laboratories). We amplified each of the seven immune genes (*lysozyme C*,* prophenoloxidase*,* Attacin*,* peptidoglycan recognition protein LC*,* ß‐1,3‐glucan recognition protein*,* serpin 3a*, and *pelle*) and three housekeeping genes (*histone variant H2A.Z*, and mitochondrial *ribosomal protein L37* and *S24*) using primers and appropriated protocols described by Woestmann et al. (Woestmann et al., 2017). For each qPCR reaction, we mixed 1 μl of the 1/5 diluted cDNA, with 5 μl of SYBR^®^ Green containing master mix (iQ™ SYBR^®^ Green Supermix, Bio‐Rad Laboratories, USA), 3 μl of nuclease‐free water, and 0.5 μl of the forward and reverse primers (10 μm). Nonreverse‐transcribed samples were used as controls for the lack of genomic DNA contamination.

### 2.7 Statistical analysis

All the statistical analyses were performed with the software R v3.3.1 (RCoreTeam, 2016).

#### 2.7.1 Larval performance

We first tested correlations among the variables using linear models (lm), from the package *lmer4* (Bates, Machler, Bolker, & Walker, 2015). As larval group size at L2 and treatment are highly correlated, only the treatment variable is used in the following models. The development time to L2 was log‐transformed prior to analysis. The development time to L2 was compared among larvae from the 27 larval families using a linear mixed model including the “treatment” as an explanatory variable and the “larval family” as a random variable. The survival rate at day13 from the 27 larval families was compared using a general linear mixed model assuming a Gamma distribution of the data, with “treatment” as an explanatory variable and the “larval family” as a random factor. We used the packages *lme4* (Bates et al., 2015) and *MASS* (Ripley et al., 2016) for the mixed model analyses. Interclass correlation coefficients (ICC) were calculated based on variance of the random factor and residual to estimate how much of the variance was explained by the random factor “larval family” in each model.

#### 2.7.2 Microbiota

We used VEGAN (Oksanen et al., 2011) in R to compute a Bray–Curtis dissimilarity matrix and analyzed bacterial composition variations among samples using nonmetric multidimensional scaling (NMDS) or distance based redundancy analysis (dbRDA) (Anderson & Willis, 2003). The α‐diversity (diversity of the microbiota within each samples) of the microbiota was estimated through the Shannon index while the β‐diversity (dissimilarity among samples) of the microbiota was estimated through the Bray–Curtis index. For the α‐diversity comparisons, a linear model was used after a logarithmic transformation of the index. The impact of the treatment, the larval family, and their interaction were considered as explanatory variables. Similarly, for the β‐diversity comparisons, we used a Permutational analysis of variance (*adonis‐*ANOVA) (Anderson, 2001) with the treatment, the larval family, and their interaction as explanatory variables.

#### 2.7.3 Metabolomics

We extracted entire spectrum values from each sample using the program MestReNova 12 (Mestrelab Research, Spain) (Willcott, 2009). For multivariate analysis, the signals were binned to 0.04 ppm, the TSP, H_2_O, and CD_3_OD signals were removed, and the integral values were transformed following the formula given below:

$$\frac{9\times \int_{\delta ‐0.02}^{\delta +0.02}\text{Intensity}}{m\times \int_{‐0.02}^{0.02}\text{Intensity of TSP}}$$

with “*m”* as the exact dry mass of the sample (±0.1 mg), “*δ”* as the ^1^H chemical shift and “9” as to the number of equivalent ^1^H atoms contained within the TSP reference molecule. Characteristic signals corresponding to α‐glucose (*δ* 4.59, d, *J* = 7.9 Hz), β‐glucose (*δ* 5.19, d, *J* = 3.7 Hz), alanine (*δ* 1.49, d, *J* = 7.2 Hz), formic acid (*δ* 8.47, s), acetic acid (*δ* 1.91, s), fumaric acid (*δ* 6.53, s), and ethanol (*δ* 1.19, t, *J* = 7 Hz) were annotated based on previously published datasets applying the same protocol (Agudelo‐Romero et al., 2014; Gogna, Hamid, & Dorai, 2015; Kim et al., 2010). *Plantago lanceolata* also contains variable quantities of iridoid and phenylpropanoid glycosides, namely aucubin, catalpol, and verbascoside (Duff et al., 1965; Marak, Biere, & Van Damme, 2000; Nieminen, Suomi, Van Nouhuys, Sauri, & Riekkola, 2003). Two characteristic peaks were identified for aucubin (*δ* 6.31, dd, *J* = 1.9 Hz) and catalpol (*δ* 6.40, dd, *J* = 1.9 Hz) based on the ^1^H‐NMR profiles of standard compounds.

We conducted a principal component analysis (PCA) including all signal bin values from 49 of the 52 larval samples. Three larval samples (larval family#1: treatment A and AR, and larval family #19: treatment R) were removed prior PCA as they clearly appeared as outliers driving most of the variation from the dataset. The first seven principal components (PCs) showed Eigenvalues > 3 and together represented over 65% of the observed variation in the PCA dataset. We analyzed the seven PCs using linear mixed models (*lmer*) (Bates et al., 2015) after log‐transformation. We included “larval treatment” as an explanatory variable and “larval family” as a random factor in each model. Interclass correlation coefficients (ICC) from each model were calculated based on the proportion of the variance explained by the random larval family factor. Tukey post hoc tests, after correction for the larval family effect using the glht function, were used to explore paired comparison between treatments. The *p‐*values were corrected for multiple testing using a Bonferroni correction (*α* = 0.025). Finally, we independently tested variations in the amount of α‐glucose, β‐glucose, alanine, formic acid, acetic acid, fumaric acid, ethanol, aucubin, and catalpol within the larvae between the treatment groups.

We tested the relationship between each of the first seven PCs and the development time to L2 and survival at day13 between the treatment groups using a linear mixed model (*lmer*), with “treatment” and the “PC” of interest as fixed factors, and “larval family” as a random factor to each model. Tukey tests after correction for the larval family effect with the glht function were used as post hoc tests to explore paired comparison between treatments. The resulting *p‐*values were corrected for multiple testing using a Bonferroni correction (*α* = 0.025).

#### 2.7.4 Immune gene expression

We calculated the mean immune gene expression from the three technical replicates (with exception of few outliers) considering the geometric mean of the three reference genes, for each larval family but one. For unknown reason, the control samples for the *S24* housekeeping gene were not expressed for the larval family#16; thus, the immune gene expression for larval family#16 was calculated based on the geometric mean of the two remaining housekeeping genes. The immune genes expression (Log2) was compared among larvae from 12 larval families using generalized linear models (Anderson, 2001; Bates et al., 2015) including the “larval treatment” and “gene” as fixed factors (including interaction term), and “larval family” as a random factor. We performed a post hoc analysis using the lsmeans function with Tukey's HSD adjustment for pairwise comparisons (Lenth, 2016), to explore paired comparison between treatments and genes, and corrected resulting *p‐*values for multiple testing using a Bonferroni correction (*α* = 0.025).

Finally, we tested whether development time to L2 (corrected for larval family effect) and survival (corrected for larval family effect) were differently affected by variation in the expression levels (Log2) of the *Attacin* immune gene (an antimicrobial peptide active against Gram‐negative bacteria (Imler & Bulet, 2005) from the different larval treatment groups, including the “immune genes expression levels” and “treatment” as explanatory variables. Tukey tests, after correction for the larval family effect using the glht function, were used as post hoc tests to explore paired comparison between treatments. The resulting *p‐*values were corrected for multiple testing using a Bonferroni correction (*α* = 0.025).

## 3 RESULTS

### 3.1 Larval performance

**FIGURE 2:**
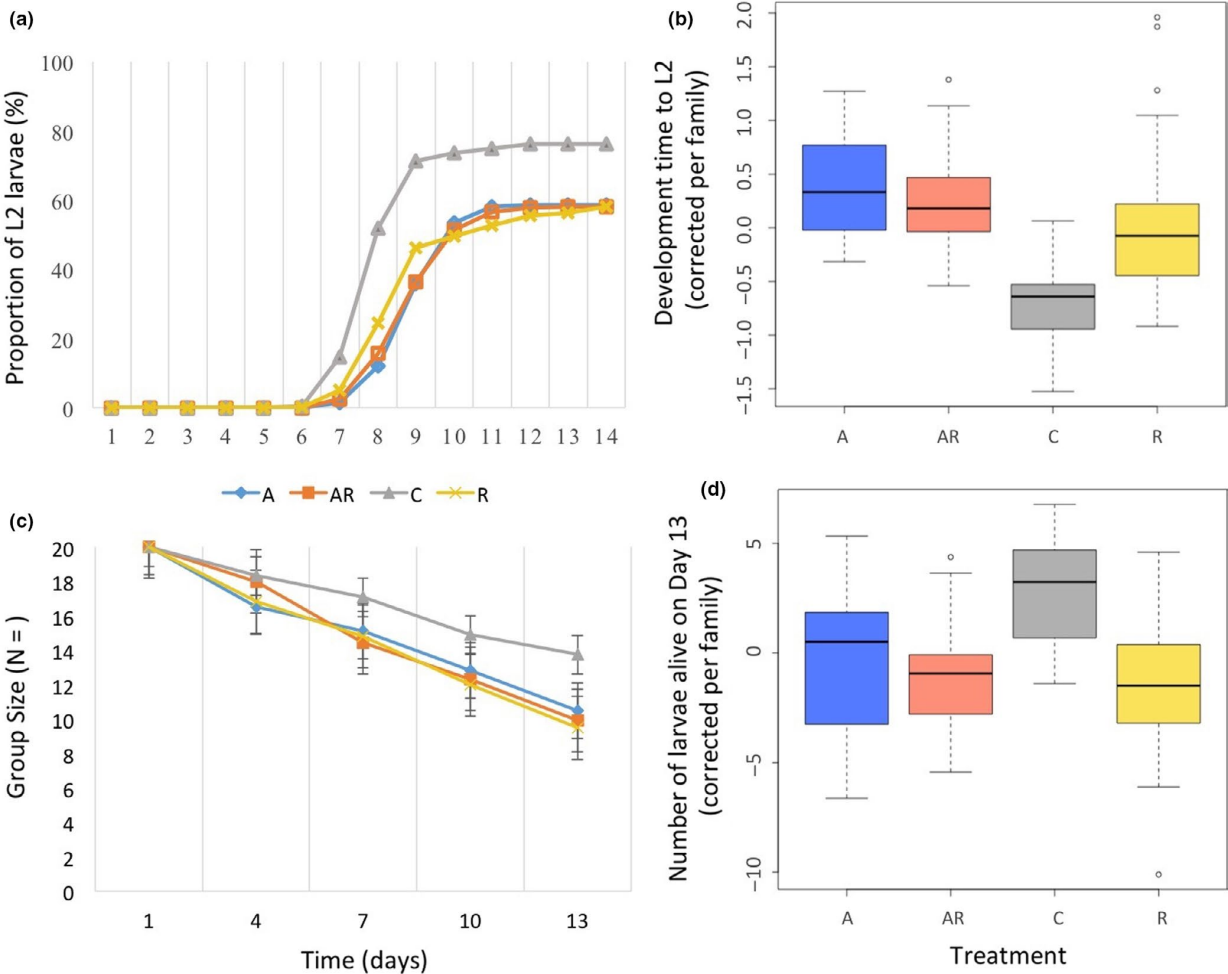
Effects of microbial depletion through antibiotic treatment on the development and survival of prediapause larvae of the Glanville fritillary butterfly. (a) Proportion of larvae from each treatment that reached L2, and (b) development time of larvae into L2 for each treatment. Data include larvae from 27 larval families under four different treatments (A‐Blue): antibiotic‐treated, (AR‐Orange): antibiotic‐treated even during re‐infected, (C‐Gray) control, and (R‐Yellow): antibiotic‐treated followed by re‐infection by L7 larval frass. (c) Survival at day 1, 4, 7, 10, and 13 after the start of the experiment, and (d) survival at day 13 for each treatment. Data include larvae from 27 larval families under four different treatments (A): antibiotic‐treated, (AR): antibiotic‐treated even during re‐infected, (C) control, and (R): antibiotic‐treated followed by re‐infection by L7 larval frass

Of the 2,160 larvae included in this study, 62.6% developed into second larval instar (L2), and 54.5% survived until L3. Larvae from the control group showed faster development (C:7.3 ± 1.9 days vs. A:8.4 ± 1.7, AR:8.3 ± 3, and R:8 ± 3.3 days, *df* = 3, *p* = 2.2e−12, Figure 2a). The ICC value of the model further suggests that 60% of the overall variance in development time to L2 was explained by performance differences among larval families (after log‐transformation, ICC = 0.60), with larval family #2 showing the longest developmental time (around 10 days), and larval family #29 showing the shortest (under 7 days). After correction for the larval family effect, developmental time remained lowest for the controls and equally high between the other treatment groups (Figure 2b).

Control groups also showed the highest survival to day 13 (L3) (C: 13.7 larvae per family group, 68.7%), while survival was equally low between the other three treatment groups (A: 10.5, 52.4%, AR: 9.9, 49.6%, and R: 9.5, 47.4% larvae per family group, Figure 2c; multiple comparison of means: Tukey contrast test *p*‐value < 7.2e−4). Note that as the larvae grew, some of them did not survive to reach L3; thus, the average larval group size also vary among the treatment groups with the control groups being the largest when larvae reach L2 (C:15.2 ± 7.2 larvae, A:11.7 ± 8.7, AR:11.6 ± 8.6 and R:11.6 ± 11.6, *p* = 2.16e−4, Figure 2c). For example, all larvae from four larval family groups died before reaching L3, two of these developed under the R treatment, one under the A treatment, and one under the AR treatment (i.e., none of the controls). Although group size at L2 varied among the treatments, we included only the treatment as an explanatory variable in the model. We do also note and discuss the potential cofounding effect of group size on larval performances. Finally, the ICC value of the model suggests that 46% of the overall variance in survival until day13 is explained by differences among larval families, with larval families #3, 19 and 20 showing the highest survival rate (>15 larvae per treatment group), and larval families #8, 24 and 25 showing the lowest (<5 larvae per treatment group; Figure S1). After correction for the larval family effect, survival to day13 remained highest for the control group and was equally low between the other three treatment groups (Figure 2d).

### 3.2 Microbiota

We independently analyzed the bacterial communities, at the phylum level only, from 156 larval samples (three L3 larva gut samples from 13/27 larval families for each of the four treatments). In parallel, we also characterized the bacterial communities of five frass samples from L7 larvae (i.e., those used to re‐infect the larvae), and five pieces of leaves from five *P. lanceolata* host plants used to feed the larvae during the experiment.

**FIGURE 3:**
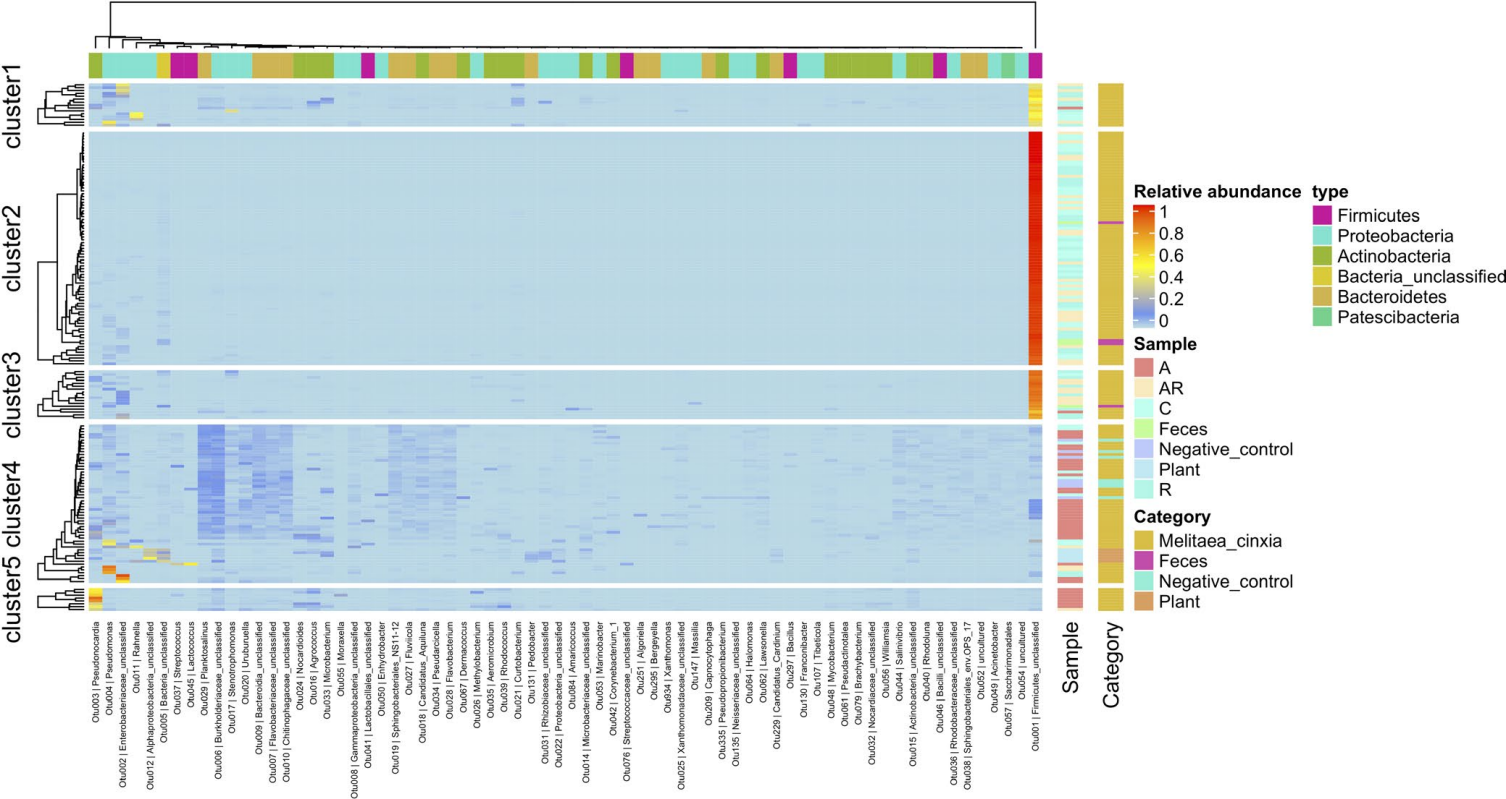
Composition of the microbiota of the host plant, the frass, and the gut of the Glanville fritillary larvae. The bacterial OTUs were reported with their taxonomical classification at the genus and phylum level (type) within the plants used to fed the larvae (Plant), the frass used to re‐infect the larvae (Feces), and within the gut of larvae from four treatment groups. (C): nontreated, (A) fed with antibiotics, (AR) fed with antibiotics while transplanted with frass, or (R) fed with antibiotics before being transplanted with frass. This dataset also includes negative controls, from blank extractions, PCR, and sequencing (negative control)

We identified 760 bacterial OTUs across all our samples (larval guts, frass, and plants). We found differences in the bacterial community composition among the treatment groups (Figure 3). The bacterial α‐diversity of the antibiotic‐treated larvae was higher to that of the other larvae (Shannon index, TukeyHSD.test, A versus AR: *df* = 5, *p* < 2e−16, A versus C: *p* < 2e−16, A versus R: *p* < 2e−16, Figure S2a), and that of the frass samples (*p* = 5.6e−6), but was similar to that of the plant samples (*p* = .96; Figure 3). The bacterial α‐diversity of the plant samples also differed from the frass samples (*p* = 1.14e−4), and of all other treatments (*p* < 6.58e−5), except the antibiotic‐treated larval samples (*p* = .96). On average, 20 bacterial OTUs per sample were characterized from the antibiotic‐treated larvae, 31 from the plant samples, 11 from the larvae of the three other treatment groups, and seven from the frass samples. The α‐diversity varied between larval families (*df* = 12, *p* = 6.03e−4), with larval family#12 showing significantly higher α‐diversity than larval family#1 (*p = *4.79e−3), #7 (*p = *1.78e−3), #9 (*p = *1.29e−3), #10 (*p = *8.77e–3), #19 (*p = *.02), and #29 (*p = *.014; Figure S3a).

Similarly, the bacterial community associated with antibiotic‐treated larvae was more heterogeneous (homogeneity of multivariate dispersion, *df* = 3, pseudo‐*F* = 7.74, *p* = .001) and differed from that characterized from the larval gut samples in the other treatment groups (*adonis*‐ANOVA, *df* = 3, pseudo‐*F* = 48.489, *R* ^2^ = 0.40, *p* = .001; Table S1). The OTUs assigned as *Planktosalinus* and unclassified Burkholderiaceae were most common in the antibiotic‐treated larvae, while unclassified Firmicutes were dominating the bacterial communities in the gut of the larvae from the other treatments (Figure 3 and Figure S4). The frass samples that were used to re‐infect the larvae showed a very similar microbial community composition to that of the larval gut of the three treatment groups (C, AR, and R) and harbored a high abundance of unclassified Firmicutes (Figure S4), thus contrasting with the microbial community from antibiotic‐treated larvae. The plant samples also did not harbor a microbial community similar to any of the other treatment groups and were mostly composed of unclassified bacteria, unclassified Alphaproteobacteria, *Pedobacter*, and unclassified Rhizobiaceae (Figure 3). Finally, 11% of the β‐diversity was influenced by the larval family factor (*adonis*‐ANOVA, *df* = 14, *F* = 2.95, *R* ^2^ = 0.11, *p* = .001; Table S1; Figure S3). It is also interesting to note that before removing the contaminating OTUs from any given sample, the antibiotic‐treated larvae harbored a similar community to that of the negative controls (blank extractions, PCRs, and sequencing; Figure 3). This observation supports the conclusion that antibiotic treatments efficiently cleared out the antibiotic‐treated larvae from their bacterial microbiota.

**FIGURE 4:**
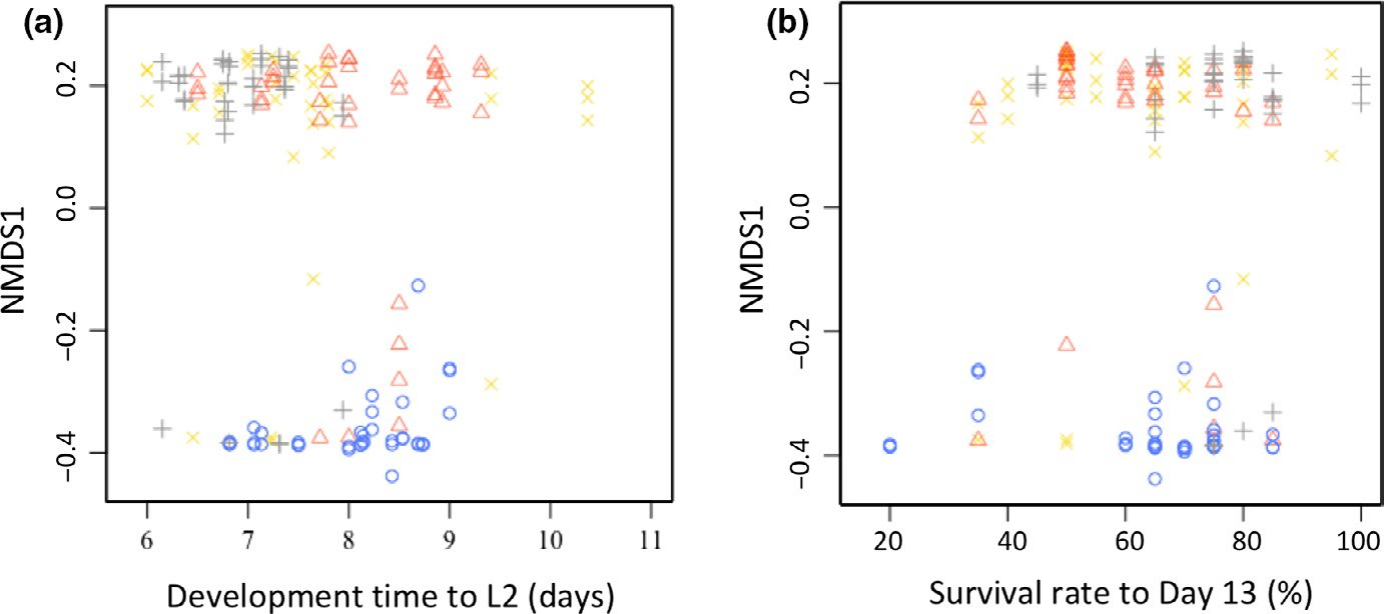
Effects of manipulation of the gut bacterial community through antibiotic treatments on the (a) development rate to L2 (days) and (b) survival rate to L3 in the Glanville fritillary larvae. Average values from three larvae from 13 families reared under four different treatments (Blue ○): antibiotic‐treated, (Orange Δ): antibiotic‐treated even during re‐infection, (Gray +): control larvae, and (Yellow ×): antibiotic‐treated followed by re‐infection by frass from L7 larvae of the same families

For the subset of 13 larval families for which both microbiota and life history data were measured, we tested whether variation of the α‐ or β‐diversity indexes correlated with variation in developmental time to L2 and survival to day13. Although we previously showed that larval developmental time to L2 varied among the treatment groups (*df* = 3, *p* = 1.26e−9), it was not correlated with variation in the diversity and composition of the gut bacterial community (Shannon index: *df* = 1, *p = *0,69; NMDS1: *df* = 1, *p* = .8; Figure 4a). Similarly, even though the larval survival to day13 varied among the treatment groups (*df* = 3, *p* = 1.08e−4), it was not associated with variation in the diversity and composition of the gut bacterial community (Shannon index: *df* = 1, *p* = .47; NMDS1: *df* = 1, *p* = .87; Figure 4b). Finally, larval family explained 55% of the variance of development time to L2 and 14% of the variance of survival in these models, respectively.

### 3.3 Metabolomics

We analyzed variation in the metabolite profile of larvae from the four treatment groups (49 larval samples) by ^1^H‐NMR. The total signals (annotated and un‐annotated) were then included in a multivariate analysis, focusing only at the first seven PCs (all PCs with Eigenvalue > 3.0) of a PCA including all metabolite data from the NMR analysis. When considering the whole metabolomics profiles, there was no effect of the treatment group on the metabolite composition of the samples (*df* = 3, *p* > .096, Table S2). Similarly, the respective variations in the amount of α‐glucose, β‐glucose, alanine, formic acid, acetic acid, fumaric acid, ethanol, aucubin, or catalpol within the larvae were not significantly affected by the treatment group (Table S3). Metabolites showed little variability across larval families (ICC < 0.001 for most PCs analyzed), except for larval family #1 which showed a different metabolome profile compared to other larval families (observed on PC6 with an ICC = 0.41, Table S2).

We further tested whether the development time to L2 and survival to day13 were correlated with the metabolite content of larvae in each treatment group. There was no significant correlation of either the larval development time to L2 (*df* = 1, *p* > .24) or survival (*df* = 1, *p* > .055; Figure S5) with any of the seven PCs describing the metabolomic profile.

### 3.4 Immune gene expression

**FIGURE 5:**
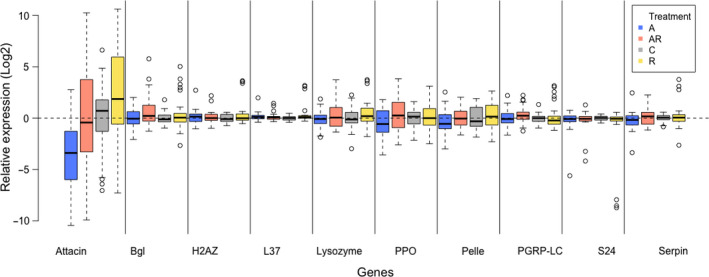
Effects of microbial depletion through antibiotic treatment on the expression levels of seven immune genes in prediapause larvae of the Glanville fritillary butterfly. Data include larvae from 12 larval families under four different treatments (A‐Blue): antibiotic‐treated, (AR‐Orange): antibiotic‐treated even during re‐infected, (R‐Yellow): antibiotic‐treated followed by re‐infection by L7 larval frass, and (C‐Gray): controls. Expression levels of the three housekeeping genes (*H2AZ*, *L37*, and *S24*) are also shown

**FIGURE 6:**
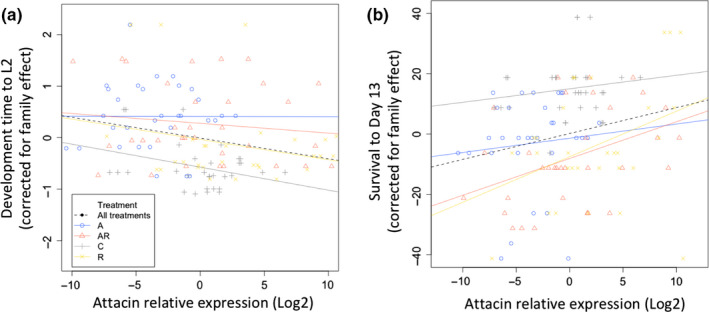
Changes in (A.) developmental time to L2 and (B.) survival to L3 with the expression levels of the *Attacin* gene in the larvae of the Glanville fritillary butterfly. Data include larvae from 12 families under four different treatments: (A‐Blue): antibiotic‐treated, (AR‐Orange): antibiotic‐treated even during re‐infected, (C‐Gray) control, and (R‐Yellow): antibiotic‐treated followed by re‐infection by L7 larval frass. The black dash line for all treatments combined

We analyzed the expression fold change (Log2) of seven immunity genes in 136 larvae from 12 larval families. Six of the seven genes did not show any consistent changes between the treatment groups (TukeyHSD.tests, *Attacin* versus any of the three control genes: *p* < 1e−4; any other comparison for other genes: *p* > .95; Figure 5). However, for *Attacin* the expression levels (corrected for larval family effect) were the lowest in the antibiotic‐treated (“A”) larvae (TukeyHSD.test, A versus C: *p* = 7.7e−3; A versus AR: *p = *3e−3; A versus R: *p* = 1.3e−6), while the expression levels of the *Attacin* gene in the “AR” and “R” samples were similar to those in the controls (TukeyHSD.test, C versus AR: *p* = .99; C versus R: *p = *.11; AR versus R: *p* = .17). Finally, once corrected for the larval family effect, the expression fold change of the *Attacin* immune gene was negatively correlated with the larval development time to L2 and positively correlated with the larval survival to day13 (*df* = 1, *F*‐value = 9.46, *p* = 2.62e−3; and *df* = 1, *F*‐value = 7.43, *p* = 7.39e−3, respectively; Figure 6, Table S4). In general, larval family ID explained over 50% of the variance in the *Attacin* gene expression level, with larval family#14 showing the highest expression levels (Data not shown).

## 4 DISCUSSION

By highlighting both the self‐resilience of the bacterial community in the gut of the larvae of the Glanville fritillary butterfly, and its consequences for the host immune homeostasis, our study contributes to the growing understanding of the complex processes that take place in the digestive tracks of Lepidoptera. We showed that the microbiota of prediapause larvae of the Glanville fritillary butterfly could efficiently be altered by antibiotic treatment and later restored to a similar composition through empirical fecal transplant. Larvae under constant antibiotic treatment (A) were successfully cleared from unclassified Firmicutes bacteria that dominate the microbiota of untreated larvae (C). In contrast, when their diet was provided with frass from conspecifics, the larvae (R) successfully recovered a gut bacterial community similar to that of their untreated conspecifics. This resilience could be the result of either a relaxation of the selective pressures induced by the antibiotic treatment or by the re‐infection process per se.

We specifically tested this by including a second control treatment (AR), during which larvae were fed on a diet treated with frass mixed with antibiotic before the relaxation of the antibiotic treatment. This particular treatment allows for testing any potential variation in the larval life histories and metabolism caused by the provision of frass only to the diet, since the frass microbiota were simultaneously removed by antibiotics. In this treatment group, the AR larvae recovered a similar bacterial species community to both the untreated and the transplanted (R) larvae. This suggests that the arrest of the antibiotic treatment, rather than the fecal transplant per se, allowed the recovery of the microbiota in these larvae. Additionally, in concordance with a study by Minard et al. (2019), that found no association between plant and larval microbiota, we showed that the bacterial species community present in the gut of control larvae was dissimilar to that associated to the host plant they fed on. Specifically, the unclassified Firmicutes we commonly found in the control larvae were undetected in the plant samples analyzed. Although Firmicutes are generally rare in the gut microbiota of other butterfly species (Chaturvedi et al., 2017; Phalnikar, Kunte, & Agashe, 2018), Ruokolainen et al. (2016) had identified a similar community structure in both field caught and laboratory reared postdiapause larvae of the Glanville fritillary butterfly, further supporting the robustness of our results. The bacterial species community characterized from the host plant leaves did, however, resemble that of the antibiotic‐treated larvae (A). One possible explanation to this may be that the treated larvae only harbor a transient microbiota, from the microbiota associated with their plant diet. Our results do not allow to fully exclude the possibility that the Glanville fritillary larvae acquire their microbiota from the environment rather than through vertical transmission, or coprophagy.

To date, evidence of transgenerational effects of the microbiota in insects is in general scarce, this is especially true for Lepidoptera. The impact of parental microbiota on the fitness of their offspring was recently tested in the large cabbage white butterfly, *Pieris brassicae* (Paniagua Voirol, Weinhold, Johnston, Fatouros, & Hilker, 2020). This study showed that altered microbial community in the mothers has no detrimental effects on the performances of their offspring when both parents and larvae fed the same host plant, but it negatively affects offspring feeding on host plants with higher sinigrin (a defensive metabolite) content than the host plant on which the mothers fed (Paniagua Voirol et al., 2020). The authors suggest that disturbances in the parental microbiota may affect the ability of their offspring to cope with the stress of host plant shift. Notably, in the Glanville Fritillary butterfly, the larval family explained a large proportion of the observed microbiota variations (11%) and of the variations in both prediapause larval development time and larval survival. The vertical transmission of symbionts has been documented in a wide range of insects, including Cockroaches, whiteflies, tsetse flies, stinkbugs, and beewolf (reviewed in Funkhouser & Bordenstein, 2013). The long‐term mutualistic relationship and the transgenerational effect of the microbiota yet remain to be characterized in the Glanville fritillary butterfly (Minard et al., 2019). In the Åland metapopulation system, the environmental conditions vary considerably geographically, and various selection pressures might act differently on the hosts and their microbial communities. For example, the spatial distribution of the two host plants of the larvae of the Glanville fritillary in the field is variable (Hanski, 2001; Hanski & Singer, 2001), and many plant species contain variable quantities of iridoid and phenylpropanoid glycosides, namely aucubin, catalpol, and verbascoside (Duff et al., 1965; Marak et al., 2000; Nieminen et al., 2003). These plant metabolites are defensive compounds known to affect the fitness of herbivorous insects (Adler, Schmitt, & Bowers, 1995; Hartmann, Theuring, Beuerle, Bernays, & Singer, 2005; Zagrobelny & Moller, 2011), including the larvae of the Glanville fritillary butterfly (Nieminen et al., 2003; Saastamoinen, van Nouhuys, Nieminen, O'Hara, & Suomi, 2007). Generally, the microbial community associated to the herbivorous insects has been thought to play a key role in the processing of such toxic compounds (Berasategui et al., 2017; Wybouw et al., 2014) and is thus of importance for insect digestion and metabolism (reviewed in Engel & Moran, 2013). However, a study performed by Minard et al. (2019) investigating field collected diapausing larvae of the Glanville fritillary butterfly showed a poor correlation between *P. lanceolata* metabolome and the composition of diapausing larval microbiota. This result is in concordance with the lack of changes in the metabolite composition we found between treatment groups. These two studies suggest that the microbiota does not impact the metabolism of larvae in the Glanville fritillary butterfly and is thus unlikely to have been locally selected to optimize local adaptation of the larval families for their host plants. Our study was however restricted to polar and highly concentrated metabolites, and other protocols might, in the future, help disentangle subtler changes in prediapause larval metabolism. Other environmental conditions, such as thermal microclimate and soil composition, may also be spatially variable. As many symbionts are predicted to be heat‐sensitive and can be eliminated or lost under thermal stresses (reviewed in Wernegreen, 2012), spatial variation in microclimates may also lead to independent selection of host families and symbiont communities.

In the Glanville fritillary butterfly, Ruokolainen et al. (2016) found a correlation between postdiapause larval growth and the host gut microbial community. They demonstrated that about 50% of the variation in postdiapause larval growth correlated with shifts in the larval gut microbial community composition and the diet of the postdiapause larvae. The larvae hosting a particular microbiota and feeding on a particular host plant species (*P. lanceolata* or *V. spicata*) were developing faster and growing larger (Ruokolainen et al., 2016). The authors suggested that diverging gut microbial communities could mediate diet‐associated differences in the larval growth of the Glanville fritillary larvae, which is known to affect their overwinter survival and postdiapause growth strategy (Saastamoinen, Ikonen, Wong, Lehtonen, & Hanski, 2013; Saastamoinen et al., 2007), as well as future adult fitness traits (Duplouy et al., 2013; Kvist et al., 2013). In our study, the larval gut bacterial community alone was not the causal reason for differences among the performance traits of the treatment groups during their early larval stages (i.e., survival and development time). Compared to the controls, a longer development time to the 2nd larval instar and a low survival rate were observed in all three other treatment groups, regardless of the composition of their gut microbiota (i.e., treatments A, AR, and R). These patterns are suggestive of a general cost of the antibiotic treatment. Chaturvedi et al. (2017) did not find any association between larval performance (using larval weight as proxy) and bacterial community composition between populations of the Melissa blue butterfly, *Lycaeides melissa*. Similarly, in *Manduca sexta*, the removal of bacteria did not affect larval weight, development nor survival (Hammer et al., 2017), and in *Danaus chrysippus* and *Ariadne merione*, the removal of the gut microbiota followed by transplants with the frass of conspecifics also did not affect any of the developmental and survival traits investigated (Phalnikar, Kunte, & Agashe, 2019). Hence, our results are consistent with the growing literature suggesting that Lepidoptera may not have a resident gut microbiota beneficial to larval growth or survival, but rather host a microbial community, which function and evolutionary importance for its host remain unclear.

Insects molt numerous times during the course of the larval development. In mosquitoes, previous studies have shown that metamorphosis and the shedding of the gut membrane led to the partial or complete renewal of the gut microbiota in the host (Moll, Romoser, Modrzakowski, Moncayo, & Lerdthusnee, 2001). The bacterial diversity has been shown to drop by 50% between the larval and the pupal stages, and to only increase again after the first feed as an adult in *Heliconius erato* butterflies (Hammer, McMillan, & Fierer, 2014). Similarly, the microbial community in *Lycaenid* butterflies is reorganized between each larval stage (Chaturvedi et al., 2017), while that of the moth *Spodoptera littoralis* partially shifts between early and late instars, and even more drastically during metamorphosis, with only *Enterococci* bacteria persisting through (Chen et al., 2016). Part of the gut microbiota of these Lepidoptera is thus voiding or cleaned from the gut lumen, suggesting that the microorganisms that may be beneficial at early developmental stages may be different from those beneficial at later developmental stages. The larvae of the Glanville fritillary butterfly go through seven to eight larval instars, a 6‐ to 9‐month‐long physiologically inactive period during the 5th instar (overwinter diapause), and metamorphoses to pupal and adult stages (Kuussaari & Singer, 2017; Saastamoinen et al., 2013). The gut environment of this species thus most likely also represents an unstable habitat for the microorganisms colonizing it. The prediapause larval stages that we studied here are potentially the most critical for the Glanville fritillary, as they show highest mortality rates in the field and the laboratory (Kahilainen, van Nouhuys, Schulz, & Saastamoinen, 2018; Kuussaari & Singer, 2017). Nonetheless, the comparison on the bacterial communities described in our study (prediapause larval stages) and in that of Ruokolainen et al. (2016) (postdiapause larval stages) showed that the gut environment of the larvae is potentially comparable at the species level throughout the different larval stages of the Glanville fritillary butterfly. Only the inactive diapausing larvae showed a different and more variable microbiota (Minard et al., 2019). Furthermore, the frass of 7th instar larvae, used in this study to transplant some of the antibiotic‐treated larvae, harbored the same communities than the gut of the control 3rd instar larvae. Nonetheless, a more comprehensive diversity comparison at the bacterial species level, and also of the absolute abundance of each bacterial species, across each of the host developmental stages should be done to confirm this observation in the Glanville fritillary butterfly. Especially, no study yet informs whether the microbiota are constantly maintained or go through bottlenecks with each molting phase, nor during metamorphosis to pupae or the adult stage in the Glanville fritillary butterfly.

The expression levels of the *Attacin* gene, which codes for an antimicrobial peptide, were down in the antibiotic‐treated larvae compared to all other treatment groups. This result suggests that the commensal microbiota are somehow involved in the permanent expression of this gene. Two potentially nonexclusive hypotheses may explain the response observed in the antibiotic‐treated larvae. First, the down‐regulation of the *Attacin* gene would suggest a relaxed stress‐response against the decrease of some bacteria from the antibiotic‐treated larvae (Asling, Dushay, & Hultmark, 1995), but present in the control and recolonized larvae. Alternatively, the host might constantly regulate the growth of its gut microbiota through the expression of the *Attacin* gene (Login et al., 2011), and the expression levels of this gene might be relaxed once all or most bacteria are removed from the gut after antibiotic treatments. The microbiota of these larvae might thus contribute to the immune homeostasis of the gut environment of this butterfly species. The up‐regulation of the *Attacin* gene in the Glanville fritillary butterfly was previously shown in larvae exposed to both bacterial and fungal pathogens (Rosa et al., 2018), in adult butterflies exposed to bacterial pathogens (Woestmann et al., 2017), and in adult butterflies after flight (Kvist et al., 2015; Woestmann et al., 2017). In our study, the expression levels of this gene were positively correlated with survival and negatively correlated with development time in the prediapause of the Glanville fritillary butterfly. Altogether, these studies suggest that in the Glanville fritillary butterfly the regulation of the *Attacin* gene is part of a response to general stress cues that benefits the fitness of the larvae.

## 5 CONCLUSION

The experimental removal of the dominant species of the gut bacterial community of the larvae significantly impacted the host immunity by down‐regulating the expression of a gene involved in the response against pathogens. Furthermore, increased expression levels of the *Attacin* immune gene were associated to improved measured life history traits (i.e., faster growth and higher survival). However, neither the life history traits nor the larval metabolism was affected by variations in the gut bacterial species community composition. Altogether, this study strongly suggests a link between the gut environment and the immune system of the Glanville fritillary butterfly. In the future, the targeted removal of microbial taxa shall further reveal the functional role of the microorganisms colonizing the gut of insects and clarify their roles in the evolution of physiological and morphological features of the host species, including their ability to cope with pathogens and other stresses.

## CONFLICT OF INTEREST

The authors declare no competing interests.

## AUTHOR CONTRIBUTIONS

**Anne Duplouy:** Data curation (equal); formal analysis (equal); funding acquisition (supporting); investigation (equal); writing–original draft (lead); writing–review and editing (equal). **Guillaume Minard:** Conceptualization (equal); data curation (equal); formal analysis (equal); investigation (equal); methodology (equal); validation (equal); writing–original draft (supporting); writing–review and editing (equal). **Marjo Saastamoinen:** Conceptualization (equal); formal analysis (supporting); funding acquisition (lead); investigation (supporting); methodology (equal); project administration (lead); validation (equal); writing‐original draft (supporting); writing–review and editing (equal).

### Open Research Badges

This article has earned an Open Data Badge for making publicly available the digitally‐shareable data necessary to reproduce the reported results. The data is available at https://doi.org/10.5061/dryad.9s4mw6mc1; http://www.ebi.ac.uk/ena

## Supporting information

Appendix S1

## Data Availability

Raw data from life history assays, qPCR runs, and NMR spectra are publicly stored in *Dryad* (https://doi.org/10.5061/dryad.9s4mw6mc1). The raw microbiota data are accessible from the European Nucleotide Archive (http://www.ebi.ac.uk/ena, European Molecular Biology Library‐European Bioinformatics Institute, EMBL‐EBI) under the project ID: PRJNA640007.
