# Supplementary material for: The gut bacterial community affects immunity but not metabolism in a specialist herbivorous butterfly
Source: Ecol Evol. 2020 Jul 16;10(16):8755–69. doi: 10.1002/ece3.6573 (PMC7452788; doi:10.1002/ece3.6573)
Supplement: Supplementary file 1 — Appendix S1 [file ECE3-10-8755-s001.docx]

**Appendices**

The ICC value of the model suggests that 46% of the overall variance in survival until day13 is explained by differences among larval families, with larval families #3, 19 and 20 showing the highest survival rate (>15 larvae per treatment group), and larval families #8, 24 and 25 showing the lowest (<5 larvae per treatment group) (Figure 1).

**A.**

**
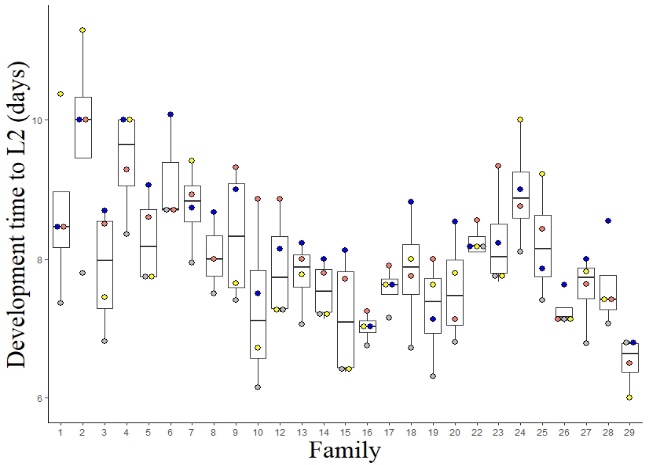
**

**B.**

**
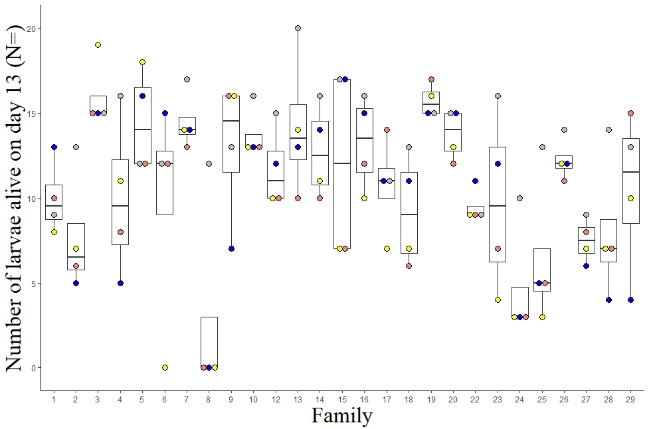
**

**Figure 1: Between larval family variations of the effect of microbial depletion through antibiotic treatment on the (A.) development rate to L2 and (B.) survival to L3 of pre-diapause larvae.** Data include larvae from 27 families under four different treatments (blue): antibiotic-treated, (salmon): antibiotic-treated even during re-infected, (gray): control, and (yellow): antibiotic-treated followed by re-infection by L7 larval frass.

The bacterial α-diversity of the antibiotic-treated larvae was higher to that of the other larvae (Shannon index, TukeyHSD.test, A vs AR: df=5, *P*<2e-16, A vs C: *P*<2e-16, A vs R: *P*<2e-16) (Figure 2).

1. B.

**
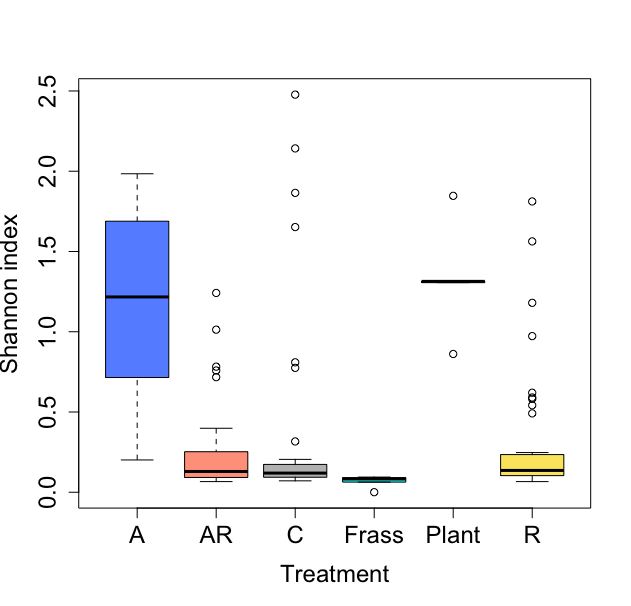
**
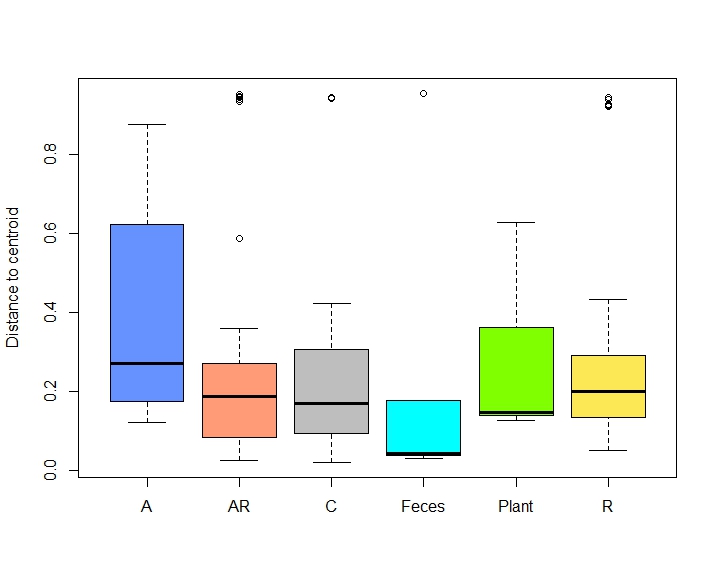


C.

**
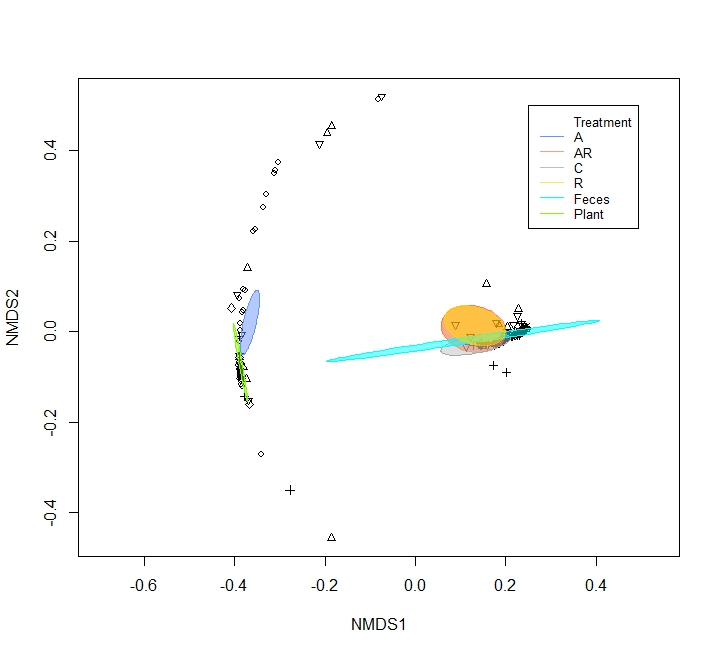
**

**Figure 2. Effects of antibiotic treatments on the (A.) α-diversity (Shannon index), (B.) heterogeneity (Distance to centroid), and (C.) β-diversity of the bacterial gut communities of pre-diapause larvae and frass of the Glanville fritillary butterfly, and of the larval host plant *Plantago lanceolata*.** Data include three larvae from each of 13 larval families, and under four different treatments: (A- squares): antibiotic-treated, (AR-circles): antibiotic-treated even during re-infected, (C-triangles): control larvae, and (R-+): antibiotic-treated followed by re-infection by frass from L7 larvae of the same larval families. (turquoise-x): frass, and (green-diamond): plant samples.

The bacterial α-diversity of the antibiotic-treated larvae was similar to that of the plant samples (*P*=0.96) (Figure 3). The α-diversity varied between larval families (df=12, *P*=6.03e-4), with larval family#12 showing significantly higher α-diversity than larval family#1 (*P=*4.79e-3), #7 (*P=*1.78e-3), #9 (*P=*1.29e-3), #10 (*P=*8.77e-3), #19 (*P=*0.02) and #29 (*P=*0.014) (Figure 3). Finally, 11% of the β-diversity was influenced by the larval family factor (*adonis*-ANOVA, df=14, *F*=2.95, R^2^=0.11, *P*=0.001) (Figure 3).

A. B.


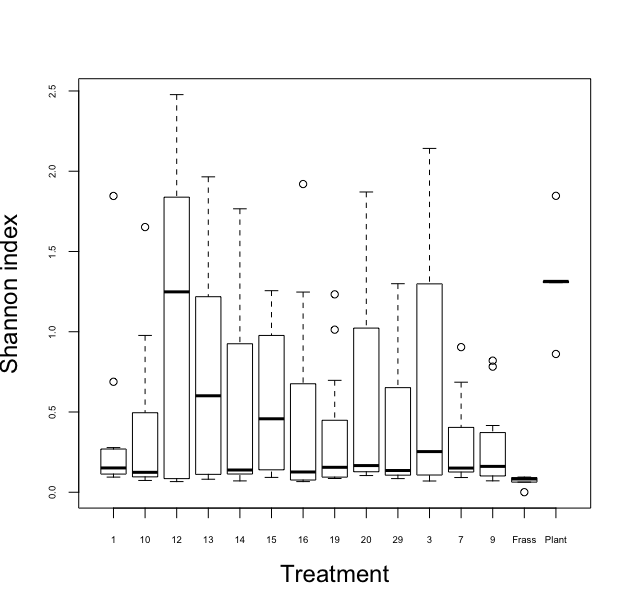

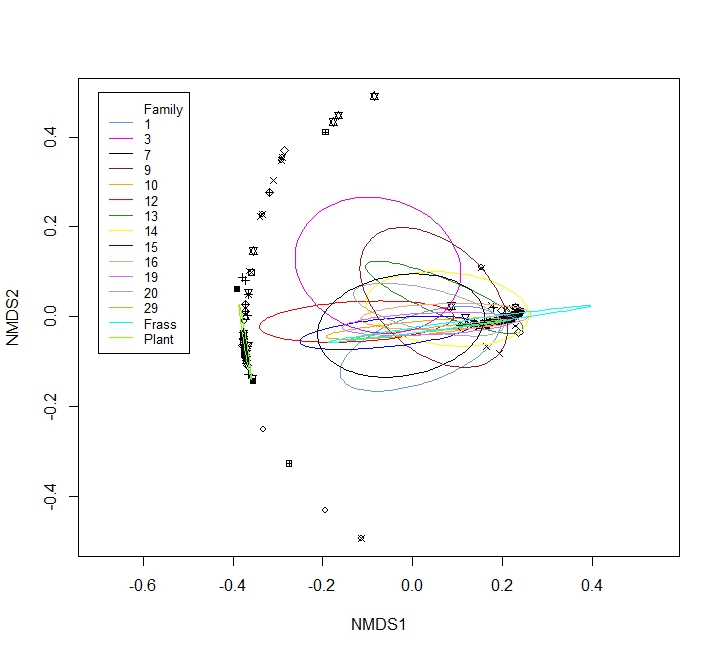


**Figure 3: Effects of larval family on the (A.) α-diversity (Shannon index) and (B.) β-diversity of the gut bacterial communities of pre-diapause larvae, frass and host plant (*Plantago* lanceolata) of the Glanville fritillary butterfly.** Data include larvae from 13 families. Bacterial communities of five larval frass samples and five host plants (1cm^2^ leave piece/plant) were added to the analysis for comparison. There is no difference between families in the bacterial community of the gut.

The frass samples that were used to re-infect the larvae showed a very similar microbial community composition to that of the larval gut of the three treatment groups (C, AR and R), and harbored a high abundance of unclassified Firmicutes, thus contrasting with the microbial community from antibiotic-treated larvae (Figure 4).

**
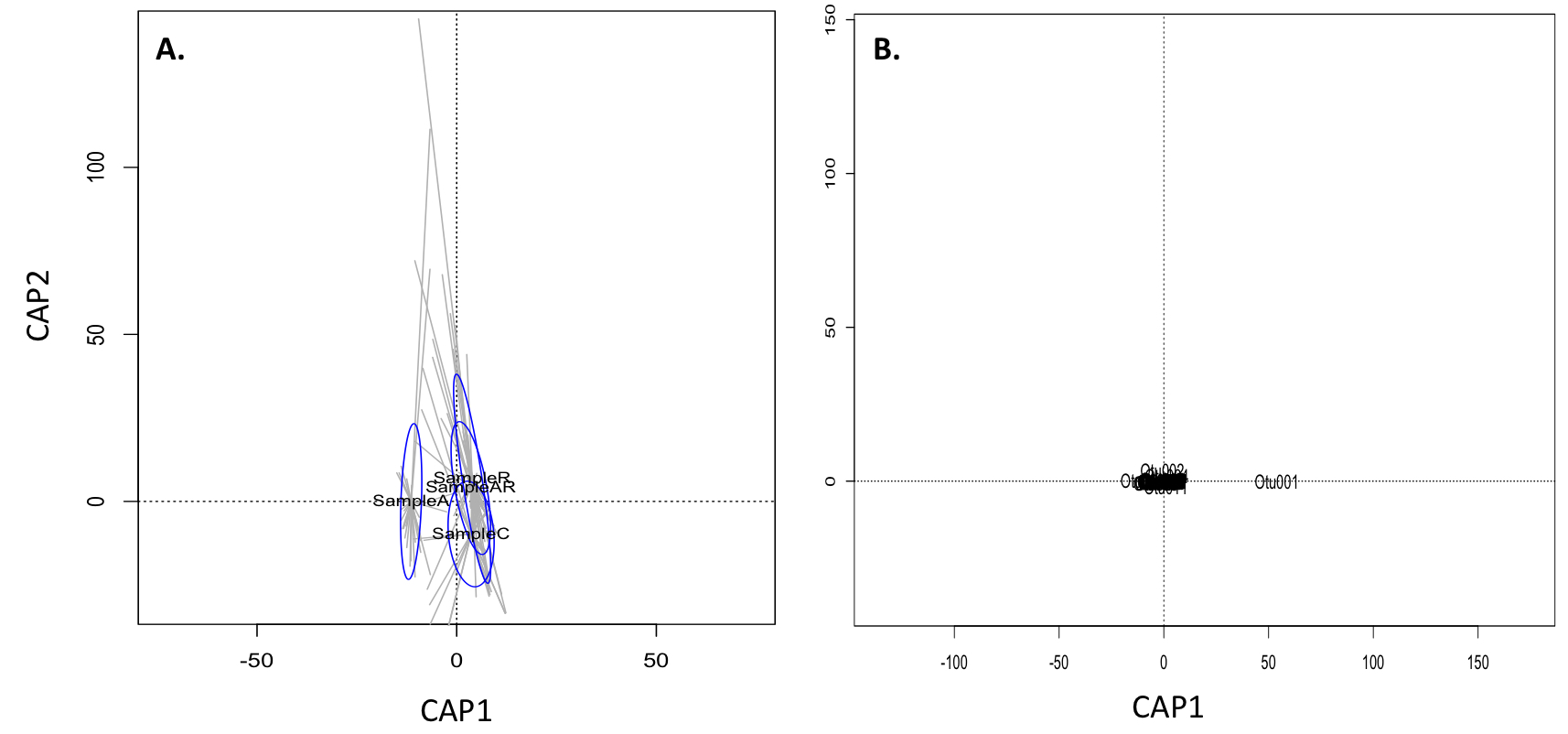
Figure 4. Distance based redundancy analysis (dbRDA) highlighting the impact of treatment on the bacterial communities in the larvae of the Glanville fritillary butterfly.** The dbRDA was performed with the Bray-Curtis distances reflecting the differences among the bacterial community of the samples. The treatments were used to constrain the analysis, which was also corrected for the larval family effect. (**A.**) The samples treatments corresponded to larvae that were either non-treated (Controls), fed with antibiotics (A), fed with antibiotics while re-infected with frass (AR), or fed with antibiotics before being re-infected with frass (R). (**B.**) The OTUs that correlated with the separation of the samples are plotted on the same axis. The Otu001 corresponds to sequences that belonged to bacteria from the Firmicutes phylum that could not be classified to any downstream taxonomical level.

There was no significant correlation of either the larval development time to L2 (df=1, *P*>0.24) or survival (df=1, *P*>0.055) with any of the seven PCs describing the metabolomic profile (Figure 5).

A. B.


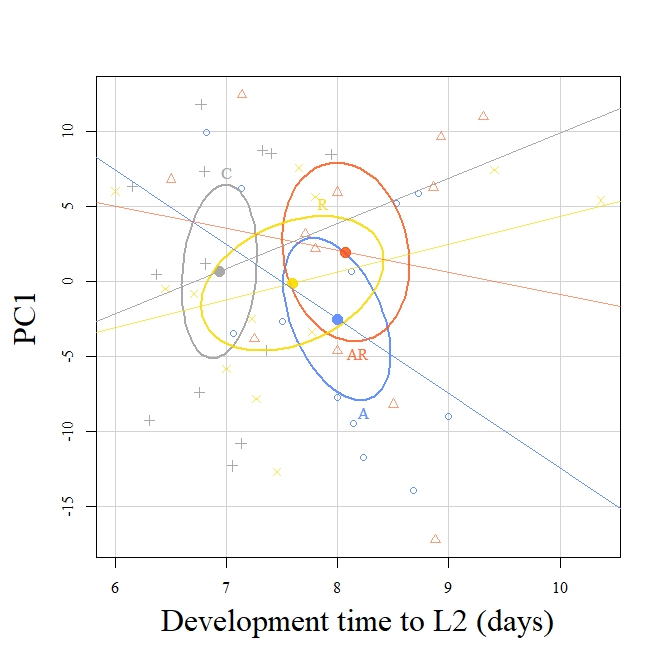

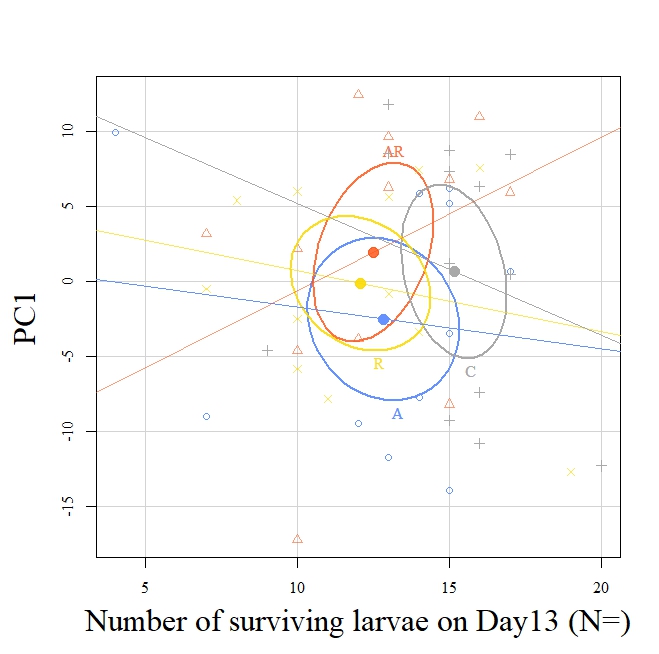


**Figure 5: PC1 values from the metabolite study according to (A) development time to L2 and (B) survival to L3 of pre-diapause larvae for each treatment group.** Average data from up to three larvae from 13 larval families reared under four treatment groups: (A-Blue): antibiotic-treated, (AR-Orange): antibiotic-treated even during re-infected, (C-Gray): control, and (R-Yellow): antibiotic-treated followed by re-infection by L7 larval frass. Ellipses give the 2% confidence interval for respective treatment group.

**Appendix Table 1. Permutational analysis (*adonis-ANOVA*) of the gut bacterial community of the larvae of the Glanville fritillary butterfly.**

| **Covariates** | **df** | **pseudoF** | **R^2^** | ***P*** |
| --- | --- | --- | --- | --- |
| **Treatment** | 3 | 48.489 | 0.40 | 0.001*** |
| **Larval family** | 14 | 2.953 | 0.11 | 0.001*** |
| **Treatment x Larval family** | 36 | 1.898 | 0.19 | 0.001*** |
| **Residuals** | 106 |  | 0.29 |  |

**Appendix Table 2. Analysis on the first seven principal components of a principal component analysis for metabolite composition of 49 samples from four larval treatments (‘A’, ‘AR’, ‘R’ or ‘C’)**. The treatment group does not explain the variation observed between samples, but larval family effect explains most of the variance between samples for PC6 only.

|  | **PC1** | **PC2** | **PC3** | **PC4** | **PC5** | **PC6** | **PC7** |
| --- | --- | --- | --- | --- | --- | --- | --- |
| **Eigen value** | 7.97 | 5.54 | 5.13 | 4.09 | 3.78 | 3.41 | 3.18 |
| **Cumulative proportion of variance** | 0.24 | 0.35 | 0.45 | 0.52 | 0.57 | 0.62 | 0.65 |
| **Treatment** | *P=*0.498 df=3 | *P=*0.169 df=3 | *P=*0.169 df=3 | *P=*0.169 df=3 | *P=*0.296 df=3 | *P=*0.096 df=3 | *P=*0.25 df=3 |
| **Larval family (ICC)** | 0.001 | 0.001 | 0.001 | 0.001 | 0.1e-16 | 0.408 | 5.9e-16 |

| **Appendix Table 3. Impact of the treatments on the abundance of nine annotated metabolites.** | | | | | |
| --- | --- | --- | --- | --- | --- |
| **Compound** | **df** | **F** | **P^1^** | | |
| α-glucose | 3,48 | 1.11 | 0.35 | | |
| β-glucose | 3,48 | 1.16 | 0.33 | | |
| Alanine | 3,36 | 2.24 | 0.10 | | |
| Formic acid | 3,36 | 0.40 | 0.75 | | |
| Acetic acid | 3,36 | 0.83 | 0.49 | | |
| Fumaric acid | 3,48 | 0.24 | 0.87 | | |
| Ethanol | 3,48 | 0.33 | 0.80 | | |
| Aucubin | 3,36 | 2.53 | 0.07 | | |
| Catalpol | 3,36 | 0.89 | 0.46 | | |
| ^1^ Corrected for larval family effect | | | |  |  |

**Appendix Table 4. Impact of the treatments, expression levels of the *Attacin* gene, and interactions, on the development time to L2 and survival to L3 of the larvae of the Glanville fritillary butterfly.** *P-*values corrected for multiple testing after Bonferroni correction (α=0.025).

|  | **df** | **F** | ***P*^1^** |
| --- | --- | --- | --- |
| **Development to L2**  *Attacin* expression level (Log2) | 1 | 9.46 | 2.62e-3 ** |
| Treatment | 3 | 11.042 | 1.95e-6 *** |
| *Attacin* expression:Treatment | 3 | 0.344 | 0.794 |
| **Survival to L3**  *Attacin* expression level (Log2) | 1 | 7.43 | 7.39e-3 ** |
| Treatment | 3 | 13.124 | 1.91e-7 *** |
| *Attacin* expression:Treatment | 3 | 0.518 | 0.671 |

^1^Corrected for larval family effect
